# Personalized Surgical Approach for Nonunion of the Second Metatarsal Fracture and Post-Traumatic Metatarsalgia: A Case Report and Literature Review

**DOI:** 10.3390/jpm15050174

**Published:** 2025-04-27

**Authors:** Roberto Bevoni, Elena Artioli, Marco Di Ponte, Silvio Caravelli, Massimiliano Mosca

**Affiliations:** 1 O.U. Orthopaedic Bentivoglio, IRCCS Istituto Ortopedico Rizzoli, 40010 Bentivoglio, Italy; marco.diponte@ior.it (M.D.P.); silvio.caravelli@ior.it (S.C.); massimiliano.mosca@ior.it (M.M.); 2 IRCCS Istituto Ortopedico Rizzoli, Via Giulio Cesare Pupilli 1, 40136 Bologna, Italy; 3 Department of Biomedical and Neuromotor Sciences (DIBINEM), Alma Mater Studiorum University of Bologna, 40123 Bologna, Italy

**Keywords:** nonunion, metatarsal fracture, metatarsalgia, pseudoarthrosis, autologous bone graft, personalized treatment

## Abstract

Nonunion of the second metatarsal presents a significant clinical challenge, often leading to pain, functional impairment, and deformity. Various treatment strategies have been described in the literature, tailored to the patient’s specific characteristics. To provide a comprehensive overview of the available therapeutic options, a literature review was conducted. In this context, this article aims to present an innovative and personalized surgical technique for patients with nonunion and an altered metatarsal formula following a proximal shaft fracture of the second metatarsal. This technique enables the simultaneous consolidation of the nonunion and restoration of metatarsal alignment, with favorable clinical, functional, and radiological outcomes observed over a three-year follow-up period.

## 1. Introduction

Fractures of the metatarsal bones are among the most prevalent injuries affecting the foot [1,2]. Among these, the fifth metatarsal exhibits the highest incidence, constituting 45–70% of all metatarsal fractures, followed by the third, second, first, and fourth metatarsals in decreasing order of occurrence [1,3,4,5]. However, when specifically considering stress fractures, the incidence pattern diverges, with the second and third metatarsals being the most commonly affected [1,3,5,6].

Metatarsal fractures can result from both direct and indirect traumas [4]. Direct trauma typically arises from crushing injuries or complex mechanisms (such as Lisfranc fracture–dislocations). Conversely, indirect trauma can stem from ankle sprains with a fixed forefoot. Furthermore, repetitive forces can induce stress fractures, a common occurrence among athletes, dancers, and military personnel [1,3,7].

Diagnosis is primarily conducted through foot X-rays. In order to evaluate intra-articular and additional bone involvement, a CT scan is advised when numerous metatarsal fractures are found [4]. An MRI can be performed if there are any suspected occult stress fractures. Additionally, ultrasound has demonstrated reliable diagnostic accuracy in such cases [8]. Nuclear medicine techniques are rarely needed [5].

The treatment of metatarsal fractures can be either conservative or surgical and should be personalized based on the patient’s specific characteristics and clinical presentation [1]. Conservative treatment typically involves immobilization in a non-weight-bearing cast for 3–6 weeks [5]. It is indicated for simple fractures and for displaced fractures of the central metatarsals limited only to the frontal plane without shortening [5]. Patients managed conservatively are typically monitored with serial radiographs, although recent studies suggest that these routine imaging follow-ups may be unnecessary, as they rarely influence treatment decisions and offer limited value in clinical management [9].

As a potential adjunct to conservative treatment in older adults with metatarsal fractures, the use of denosumab has also been proposed. Although based on the limited evidence of a recent case report, it was observed that denosumab may have a positive effect on fracture healing, possibly resulting in delayed remodeling. However, further research is definitely needed to validate these preliminary findings [10].

Surgical intervention, on the other hand, is reserved for fractures displaced in the sagittal plane and fractures of the first and fifth metatarsals displaced in the transverse plane [3,5]. Reduction is recommended when there is at least a 3–4 mm displacement or a 10° angulation [5]. Following reduction, fixation methods such as K-wires, screws, plates, external fixators, or tension band wiring are employed for stabilization, with the choice of fixation tailored to the specific needs of each patient [3,5]. The objective of surgical intervention is to restore both length and alignment along the sagittal plane [3].

Metatarsal fractures generally have a favorable prognosis, with most cases healing uneventfully through conservative or surgical management. However, certain complications have been reported, which can significantly impact functional outcomes [4]. Among these, nonunion is a well-documented issue, particularly in fractures involving the proximal diaphysis of the fifth metatarsal, where biomechanical factors and limited vascular supply contribute to impaired healing. Additionally, post-traumatic deformities may arise, leading to alterations in foot biomechanics that can disrupt proper load transmission during gait. Such deformities can also result in painful prominence, creating discomfort and difficulties with footwear, ultimately affecting the patient’s daily activities and quality of life [3,7,11,12].

This article aims to provide a comprehensive and detailed overview of all available techniques for the treatment of second metatarsal nonunion, carefully analyzing their indications, advantages, and limitations on a case-by-case basis. Given the complexity and variability of nonunion presentations, treatment must be tailored to the specific needs of each patient, taking into account factors such as fracture location, degree of bone resorption, presence of deformity, and the patient’s functional demands. In this context, the article also introduces an innovative and highly customizable surgical technique designed to address nonunion while simultaneously correcting associated post-traumatic deformities. Furthermore, to illustrate the clinical application and effectiveness of this personalized strategy, a detailed case report is presented. The case involves a patient with nonunion of a second metatarsal fracture associated with post-traumatic deformity, a complex scenario requiring an individualized treatment plan to achieve both bony consolidation and anatomical realignment. Through this case, the article highlights the importance of a patient-centered surgical approach, demonstrating how a customized technique can lead to successful clinical, functional, and radiological outcomes.

## 2. Case Report

A 53-year-old female patient sustained a traumatic fracture of the second metatarsal in her left foot. Apart from being a smoker, which is a well-known risk factor for impaired bone healing, she had no significant medical history, with no known metabolic disorders, osteoporosis, or other conditions that could have affected fracture consolidation. The initial management of the fracture was non-operative and was carried out at a different institution, where the patient was treated with immobilization in a plaster cast for a total duration of five weeks.

During the first three weeks, strict non-weight-bearing was advised to minimize mechanical stress on the fracture site and allow for initial healing. After this period, the patient was instructed to gradually transition to partial weight-bearing, followed by progressive functional rehabilitation of the foot and ankle. This re-education phase included gentle range-of-motion exercises, proprioceptive training, and progressive loading to restore strength and mobility while minimizing the risk of complications such as stiffness or muscle atrophy.

Despite adherence to the prescribed conservative treatment plan, the patient continued to experience persistent pain and functional limitations, raising concerns about the adequacy of fracture healing. Clinical follow-up and imaging assessments were subsequently performed to evaluate fracture consolidation and detect any potential complications, including delayed healing or nonunion.

Therefore, following conservative treatment, the patient underwent surgical intervention with open reduction and internal fixation using two screws three months later at the same institution because of persistent symptoms. However, she continued to experience persistent pain and chronic swelling of the left forefoot, prompting her to seek evaluation at our institute 2 years after the initial trauma. Physical examination revealed tenderness at the proximal diaphysis of the second metatarsal, widespread swelling of the forefoot, and transfer metatarsalgia involving the 3rd and 4th metatarsals of the left foot.

**Figure 1:**
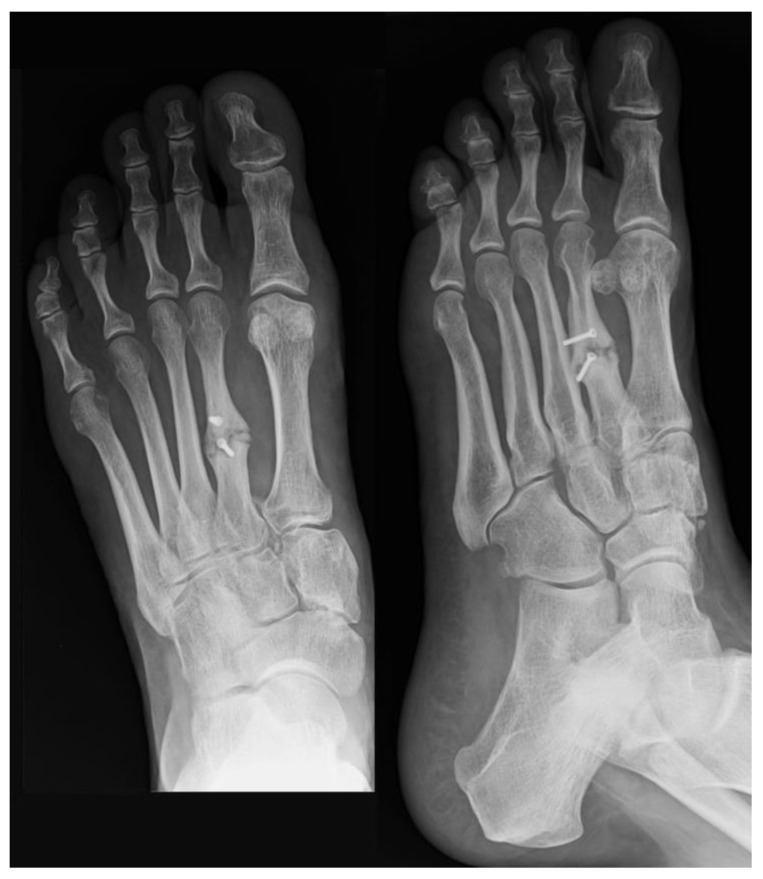
Weight-bearing dorsoplantar X-rays of the left foot revealing nonunion of the second metatarsal and a mild alteration in the metatarsal formula.

The subsequent X-rays displayed nonunion of the second metatarsal with associated slight metatarsal formula alteration (Figure 1).

A CT scan conducted two years after the initial trauma revealed the absence of consolidation and loosening of the screws. The patient was thus diagnosed with pseudoarthrosis of the second metatarsal along with an associated post-traumatic deformity, leading to the scheduling of a surgical intervention.

### Surgical Procedure

The surgical procedure was performed under spinal anesthesia by an experienced foot and ankle surgeon, ensuring optimal intraoperative analgesia and postoperative pain control. The patient was placed in a supine position on the operating table, with the affected limb properly supported and prepped in a sterile fashion. A pneumatic tourniquet was applied at the root of the thigh and inflated to ensure a bloodless surgical field, allowing for better visualization of the anatomical structures and precise handling of the nonunion site.

A dorsal longitudinal incision was made along the second metatarsal, carefully planned to provide adequate exposure while minimizing soft tissue trauma. Special attention was given to preserving the surrounding vasculonervous structures, particularly the first and second dorsal metatarsal arteries, which run anteriorly over the corresponding dorsal interossei muscles, to reduce the risk of postoperative neurovascular complications.

The extensor digitorum brevis and longus tendons of the second toe were retracted laterally to expose the bony surface. Upon accessing the site of pseudoarthrosis, meticulous debridement was performed to remove fibrous and necrotic tissue, facilitating the preparation of a viable bone interface for subsequent reconstruction.

The two previously implanted screws, which were no longer providing stability, were carefully removed. To address the nonunion, the compromised segment of the second metatarsal was excised through two parallel oblique osteotomies, ensuring that only healthy, well-vascularized bone remained. The osteotomy planes were planned to optimize contact between the bone surfaces, enhancing the potential for biological healing.

Following bone resection, the medullary canal of the second metatarsal was opened using a 2 mm diameter K-wire, a crucial step to improve the receptivity of the bone for grafting material and promote intramedullary revascularization. This preparation facilitated the next phase of the procedure, which involved reconstruction with a technique specifically tailored to the patient’s anatomical and biomechanical needs.

**Figure 2:**
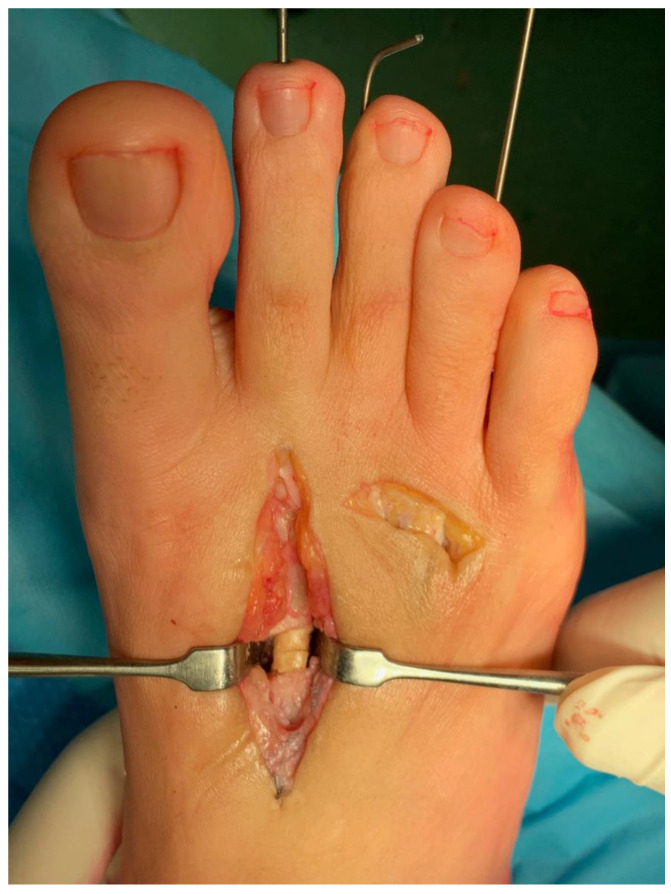
The two bone cylinders harvested from the third and fourth metatarsals used as autografts at the pseudoarthrosis site.

Subsequently, through a distal dorsal approach, a retrocapitatum oblique cylindrical osteotomy was performed to shorten the third and fourth metatarsals for realignment of the metatarsal formula. Autologous bone cylinders harvested from the third and fourth metatarsals were used as autografts at the pseudoarthrosis site (Figure 2). It is crucial to execute all osteotomies with consistent obliquity to facilitate optimal alignment of the bony surfaces.

**Figure 3:**
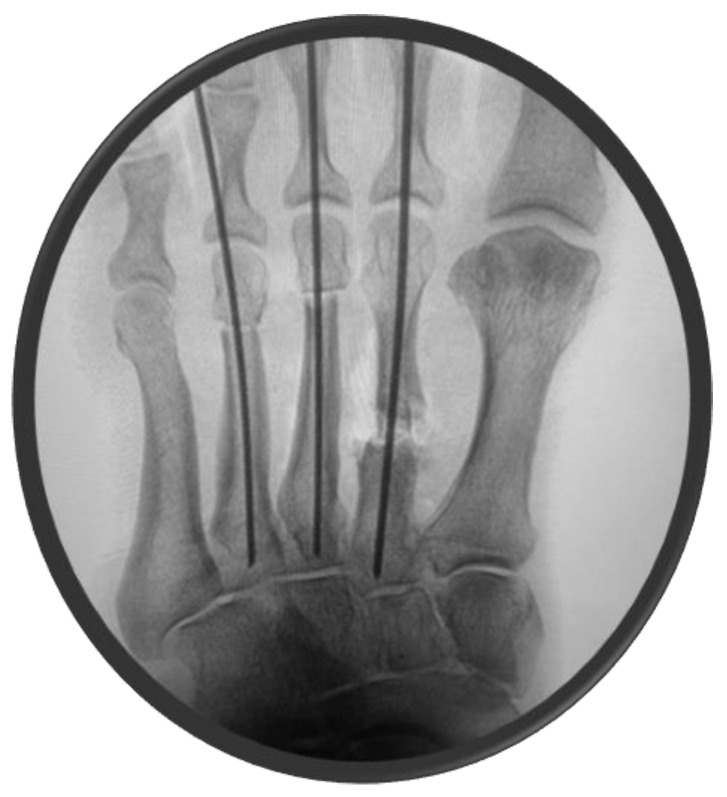
Fluoroscopic control post-synthesis using 1.4 and 1.6 K-wires.

The second metatarsal was synthesized using a 1.6 mm K-wire, whereas the third and fourth metatarsals were stabilized using 1.4 mm K-wires emerging from the base of the apex of the second, third, and fourth rays under fluoroscopic control (Figure 3).

The tourniquet was released to achieve meticulous hemostasis, and the wounds were closed with interrupted sutures using absorbable threads. A forefoot dressing was applied and kept in place permanently for 5 weeks.

**Figure 4:**
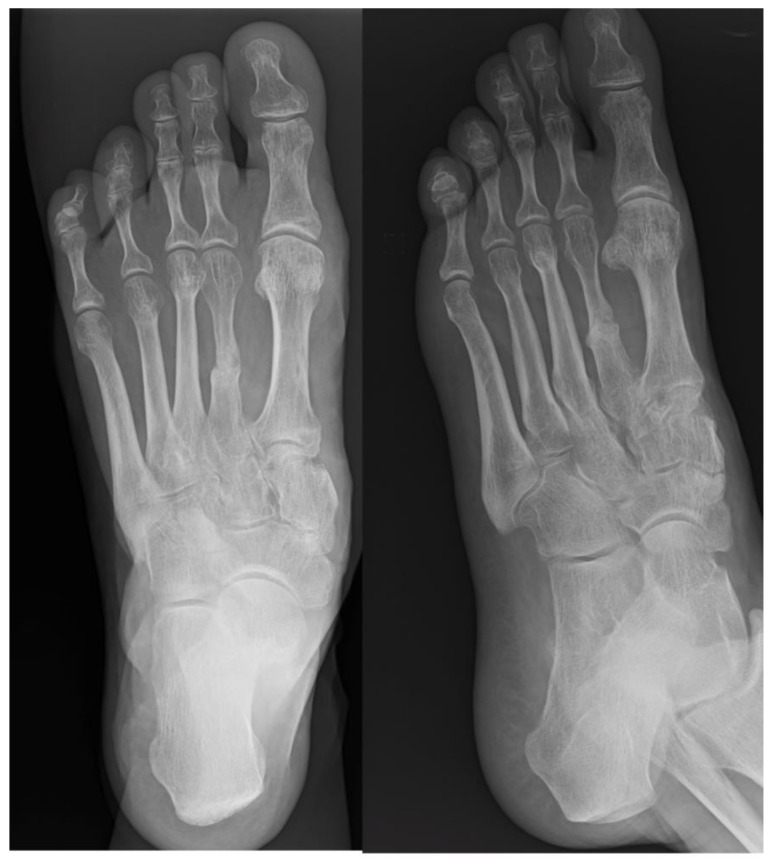
Postoperative weight-bearing X-rays at 8 months showing a solid and well-aligned union of the second metatarsal and restoration of the correct metatarsal formula.

Postoperatively, ambulation was allowed using a heel weight-bearing shoe for 5 weeks. After this period, suture removal and removal of K-wires were performed, followed by progressive weight-bearing with crutches. At 8 months post-surgery, radiographs revealed a solid and well-aligned union and restoration of the correct metatarsal formula (Figure 4), accompanied by a significant clinical improvement. No complications occurred.

At the latest 3-year follow-up, the patient remained asymptomatic, exhibited no functional limitations, and walked unaided using regular commercially available footwear.

The patient granted consent for the publication of the case study.

## 3. Literature Review

Nonunions and refractures can be linked to biological insufficiency, inadequate reduction or fixation, and excessively aggressive postoperative rehabilitation [13]. Regarding metatarsals, nonunions are most frequently observed in Jones fractures, with reported incidences reaching up to 11% [14,15,16,17].

Nonunion of the second metatarsal is a rare occurrence that has been infrequently reported in the literature. A comprehensive review conducted on this subject revealed only seven articles discussing this specific condition, highlighting its relative rarity. The majority of the available literature consists of case reports or small case series, which offer valuable insights into the management of this challenging issue but lack large-scale studies or randomized trials to establish more universally applicable treatment protocols. These reports provide detailed descriptions of individual cases, presenting a variety of clinical presentations, treatment strategies, and outcomes.

The surgical techniques employed to address second metatarsal nonunion vary considerably across the studies. These variations include differences in the type of fixation devices used, which range from K-wires and screws to locking plates and external fixation devices. Additionally, the fixation methods adopted in these studies differ, with some utilizing primary fixation, while others incorporate additional strategies, such as bone grafting, to enhance the healing process and address any underlying bone defects. In cases where bone grafting is applied, the choice between autogenous or allogenic grafts also varies depending on the patient’s individual needs and the surgeon’s preference. Despite the limited number of articles available on this topic, they provide a broad spectrum of treatment options, emphasizing the importance of individualized care in managing metatarsal nonunions.

This review aims to provide a comprehensive and in-depth examination of the various treatment strategies available for the management of metatarsal nonunions, with a specific emphasis on personalized, patient-centered approaches. Metatarsal nonunions present unique challenges, as the optimal treatment method must be tailored to the specific characteristics of each patient, including the location and type of fracture, the patient’s activity level, age, underlying health conditions, and overall functional demands. The review highlights the importance of assessing these factors in the decision-making process, as a one-size-fits-all treatment plan is often insufficient for achieving successful outcomes. By highlighting specific case reports and surgical techniques, this review illustrates the practical application of personalized treatments in real-world clinical settings, demonstrating how a customized approach can lead to more favorable results for patients with metatarsal fractures and nonunions.

A comprehensive overview of the surgical techniques described in the literature is presented below.

### 3.1. Plate

Zanovello et al. reported a case involving a 10-year-old child who sustained a fracture of the proximal shaft of the second metatarsal. The initial fracture management approach was not specified in detail, but due to complications in the healing process, surgical intervention was deemed necessary. After a prolonged period of conservative treatment, which lasted for 10 months without achieving satisfactory bone consolidation, the patient exhibited signs of delayed union or nonunion, prompting the need for operative fixation.

The surgical procedure involved open reduction and internal fixation using a plate and screws to achieve stable osteosynthesis. This technique was selected to provide rigid fixation, ensuring proper alignment and promoting secondary bone healing. Given the child’s age and skeletal immaturity, careful planning was required to minimize disruption to the growth plates and preserve normal foot development. At the two-year postoperative follow-up, a favorable clinical outcome was reported [11].

### 3.2. Compression Plate and Bone Graft

Muscolo et al. reported the case of a 24-year-old ballerina who developed a nonunion following a stress fracture at the proximal metaphyseal–diaphyseal junction of the second metatarsal. The fracture, which had initially been managed conservatively, failed to heal over an extended period, resulting in the development of nonunion. Given the nature of the injury and the patient’s active lifestyle, surgical intervention was considered the most appropriate course of action to restore both bone integrity and function.

The patient underwent surgical treatment using a low-contact dynamic compression plate. To enhance bone healing and ensure optimal stability, an autogenous corticocancellous inlay bone graft was utilized. This graft was carefully placed at the nonunion site to promote osteointegration and facilitate the healing process. The procedure was performed 10 months after the initial fracture, following the failure of conservative management to achieve fracture consolidation.

Postoperatively, the patient was closely monitored to assess the progress of healing. Over the course of the recovery period, the authors noted a successful outcome. Remarkably, the patient was able to achieve a full return to dancing within three months after the surgical intervention, demonstrating the effectiveness of the surgical approach and the relatively quick recovery that can be achieved with appropriate management of nonunion in athletic individuals [18].

### 3.3. Bridging Plate and Bone Graft

Morio et al. reported the case of a 19-year-old soccer player who developed a nonunion of a stress fracture at the base of the second metatarsal. The patient was an active athlete, and the initial conservative management over the following months failed to result in adequate healing, leading to nonunion. Given the patient’s young age, athletic demands, and the failure of conservative treatment, surgical intervention was deemed necessary to restore the structural integrity of the metatarsal and facilitate a return to sports.

The surgical approach utilized in this case involved autogenous cancellous iliac bone grafting, a technique selected to enhance bone healing due to the graft’s ability to provide both osteogenic and osteoinductive properties. The cancellous bone graft was carefully harvested from the iliac crest, providing the necessary material to fill the nonunion site and promote osteogenesis. In addition to the bone grafting, plate fixation was employed to stabilize the fracture and maintain alignment during the healing process. However, due to the size and shape of the proximal metatarsal fragment, which was deemed too small to allow direct fixation, a locking plate was used to bridge the second metatarsal to the medial cuneiform, creating a stable construct that also helped to restore the proper alignment of the foot.

The surgery was performed six months after the initial fracture, at a point where nonunion had been firmly established and the patient’s condition was not improving with conservative methods. Postoperatively, the authors reported that the patient successfully returned to sports four months after the surgical intervention. This recovery timeline underscores the effectiveness of the combined approach of autogenous bone grafting and locking plate fixation in addressing nonunion, enabling a relatively quick and successful return to high-demand activities such as soccer [19].

### 3.4. T-Type Miniplate, Interfragmentary Screws, and Bone Graft

Chuckpaiwong et al. reported their experience with five cases of nonunion following stress fractures at the base of the second metatarsal in non-dancers, a relatively rare occurrence. All of these cases were managed surgically, as conservative treatment had failed to achieve adequate fracture healing. The management strategy included careful reduction of the fracture site, followed by osteosynthesis to stabilize the bone and promote healing. To achieve this, the authors used a T-type miniplate in combination with interfragmentary screws, which provided sufficient stabilization of the fracture.

In addition to fixation, bone grafting was utilized to enhance the healing process. The type of bone graft used varied between patients: three received autogenous bone grafts, while two patients were treated with allogeneic grafts. This combination of fixation and bone grafting aimed to address the nonunion and facilitate proper bone healing. However, despite these efforts, one patient who had received an allograft experienced persistent nonunion and, as a result, underwent revision surgery. During the revision procedure, the fixation was upgraded to a more robust construct, using a 1/4 tubular plate and screws that extended to the cuneiform to provide additional support and stabilization for the nonunion site.

Unfortunately, the donor site for the autologous bone grafts used in three of the patients was not specified in the report, leaving this detail unclear. Nevertheless, this study provides valuable insights into the surgical management of metatarsal nonunion, illustrating the complexity and potential challenges of treatment, as well as the variability in patient responses to different grafting strategies [20].

### 3.5. Drilling

Sarimo et al. published a case series documenting eight instances of nonunion following the failed conservative treatment of stress fractures located at the junction between the diaphysis and metaphysis of the proximal second metatarsal. These fractures were not able to heal adequately with conservative approaches, leading to the development of nonunion in these patients.

The surgical approach used in these cases involved drilling around the fracture line, a technique designed to promote bone healing in the region of the nonunion. Following the surgical intervention, the patients were closely monitored through periodic radiographic imaging to assess the progress of bone healing. Radiographic evidence of healing was observed in all cases within a time frame of 2 to 4 months after the procedure.

Furthermore, the results of this study demonstrated a successful functional outcome, with eight out of the nine patients returning to their pre-injury activity levels within 4 to 6 months postoperatively. This suggests that, with the appropriate surgical intervention, the vast majority of patients were able to recover fully and regain their ability to perform their usual physical activities [21].

### 3.6. Resection

Micheli et al. described a case of nonunion involving a proximal fracture of the second metatarsal, which impacted the volar–medial aspects of Lisfranc’s joint. This fracture also included a necrotic fragment, further complicating the healing process. Given the complexity of the injury and the failure of conservative treatments, the patient underwent surgical intervention. The surgical procedure consisted of the resection of the necrotic fragment to remove the compromised tissue.

Following this intervention, the patient’s progress was closely monitored, and at a follow-up of 3.5 years after the surgery, the patient had not experienced any recurrence of symptoms. Importantly, the patient had successfully returned to dancing, demonstrating a full recovery and a return to pre-injury activity levels [22].

### 3.7. Bone Graft

Morris et al. reported on four cases of nonunion, two of which were asymptomatic and did not require any intervention. The other two cases, however, required surgical bone grafting to address the nonunion and promote healing. Unfortunately, this study did not specify the type of fixation devices used during the surgical procedure, leaving this aspect of the treatment plan unclear. Furthermore, the origin of the bone grafts utilized in surgical interventions was not disclosed. Another important detail that was not addressed in this study was the timing of the surgical intervention in relation to the initial trauma, which could have provided further context on the duration of nonunion before surgery and its potential impact on the final outcomes. Despite these limitations, this study still contributes to the understanding of nonunion management, especially regarding the variation in treatment needs depending on the presence or absence of symptoms [23].

## 4. Discussion

The cases previously cited in the literature review primarily involve fractures located at the base or metadiaphyseal region of the second metatarsal. In contrast, the present case concerns a fracture of the proximal diaphysis. This may explain why nonunion without concurrent malunion was the complication most commonly reported in the reviewed literature, whereas malunion was present in our case report, leading to marked metatarsalgia. This difference may be due to the strong ligamentous attachments in the proximal part of the second metatarsal, which help preserve alignment, particularly in the sagittal plane. However, as the fracture site moves more distally, the likelihood of displacement increases. Head and neck fractures are more susceptible to displacement due to the traction of the flexor tendons. In contrast, diaphyseal fractures generally remain stable as long as the interosseous and lumbrical muscles and surrounding ligaments remain intact [24]. Nevertheless, in the present case, although the fracture was diaphyseal, it exhibited sufficient displacement to result in symptomatic metatarsalgia.

In this specific case report, a nonunion developed in a proximal shaft fracture of the second metatarsal, initially managed conservatively but later converted to surgical intervention due to delayed healing. The patient, a middle-aged woman, had smoking as the sole identifiable risk factor that could compromise bone healing.

The fracture, being a simple diaphyseal fracture without association with adjacent fractures, was initially considered suitable for conservative treatment with immobilization. The conservative treatment involved immobilization for a duration of 5 weeks, with progressive weight-bearing permitted starting from the third week.

However, it is crucial to consider that the vascularization of the proximal shaft of the second metatarsal is situated within a critical zone, precisely at the intersection of two distinct blood supply transitions. Therefore, a fracture occurring in this location takes place in a particularly sensitive area [11]. Nevertheless, in the existing literature, there are no specific distinctions regarding the conservative management to be adopted based on the location of fractures in the central metatarsals. Therefore, the initial conservative protocol for this patient aligned with current recommendations [3,5].

Consequently, the occurrence of nonunion in this patient was likely influenced by smoking, coupled with a genetic predisposition, and perhaps a potential initial underestimation of the severity of the fracture.

In addition to nonunion, another complication of inadequate healing of metatarsal fractures is the development of post-traumatic deformities. Reduction is recommended in all fractures with shortening and/or displacement in the sagittal plane, and for the first and fifth metatarsals also in the transverse plane [1,4]. Surgery is necessary when closed reduction is not feasible and when the fracture stability cannot be ensured. An altered metatarsal formula and deviations greater than 20° are hardly tolerated by patients who may develop transfer metatarsalgia [3,11,25].

Despite undergoing open reduction and internal fixation, this patient experienced both complications. Specifically, the patient not only developed nonunion of the fracture but also encountered metatarsalgia due to the shortening of the second metatarsal. Therefore, the goal of the surgical treatment was to address both complications, providing a biological stimulus, ensuring the stability of the fracture to facilitate bone healing, and restoring the axis and length of the metatarsal to reestablish the metatarsal formula.

In the scientific literature, various methods for treating nonunion have been described, with autologous bone grafting being considered the gold standard [26]. Different donor sites have been mentioned, such as the iliac crest, calcaneus, and distal tibia [13]. However, it should always be considered that the use of autografts might result in donor site morbidity, with some authors even preferring to avoid it [11,26,27].

In this case report, autologous bone grafts were utilized and consisted of bone cylinders harvested from the shortened third and fourth metatarsals to restore the correct metatarsal formula, without the need to involve other donor sites.

This personalized approach allowed for both resolving the nonunion and restoring the metatarsal formula in a single procedure without further harm to the patient.

It therefore appears to be highly effective in the treatment of metatarsal nonunions. Moreover, given the psychological burden often associated with nonunion, arising from multiple surgical interventions, recurrent hospitalizations, and enduring physical and functional disabilities, a single therapeutic approach directed towards the comprehensive resolution of the clinical scenario could significantly enhance the quality of life for affected patients [28].

This study presents certain limitations. Firstly, it is centered on a single-patient case report, which inherently limits the applicability of the results to broader patient populations. Secondly, nonunions of the second metatarsal are infrequently documented in the literature. As a result, the existing literature is highly case-specific, and there is significant variability in the surgical techniques described, posing a challenge to the development of standardized treatment guidelines.

## 5. Conclusions

Nonunions of the second metatarsal are rarely reported in the literature, making them a less common yet challenging clinical issue. This review highlights that surgical treatments for these nonunions are extremely varied and must be tailored to the individual patient. Factors such as the fracture’s location, the patient’s overall health, activity level, and the presence of any associated deformities must be carefully considered.

Moreover, despite the inherent limitations of a single-patient case report, the described surgical technique appears to be a promising solution, particularly when considering the individualized nature of the treatment plan. In fact, this approach stems from the need to address both the complications present in the patient and to provide a personalized therapeutic strategy tailored to her specific needs.

Existing literature supports the efficacy of nonunion treatment using autologous bone grafts and appropriate fixation methods, which can be adjusted based on the patient’s unique circumstances. In the described technique, successful intraoperative stability was achieved through K-wire fixation. Furthermore, the use of grafts harvested from adjacent metatarsals, aimed at restoring the appropriate metatarsal formula simultaneously, represents a dependable and replicable solution for central metatarsal nonunions. This technique is particularly advantageous in addressing both the nonunion itself and associated deformities in a way that restores normal metatarsal alignment and function. Ultimately, this personalized approach offers high potential for successful outcomes while ensuring that the treatment is optimally suited to the patient’s unique anatomical and functional needs.

## Data Availability

The data presented in this study are available upon request from the corresponding author due to privacy, legal, and ethical reasons.
